# The chromosomal genome sequence of the sponge *Cliona cf. orientalis *Thiele (1900) and its associated microbial metagenome sequences

**DOI:** 10.12688/wellcomeopenres.24304.1

**Published:** 2025-07-09

**Authors:** Emma Marangon, Blake D. Ramsby, Heidi M. Luter, Sara C. Bell, Patrick Laffy, Nicole S. Webster, Ute Hentschel, Cara Fiore, Graeme Oatley, Torsten Thomas, Elizabeth Sinclair, Eerik Aunin, Noah Gettle, Camilla Santos, Michael Paulini, Haoyu Niu, Victoria McKenna, Rebecca O’Brien, Wellcome Sanger Institute Tree of Life Management, Samples and Laboratory Team, Wellcome Sanger Institute Scientific Operations: Sequencing Operations, Wellcome Sanger Institute Tree of Life Core Informatics Team, EBI Aquatic Symbiosis Genomics Data Portal Team, Aquatic Symbiosis Genomics Project Leadership

**Affiliations:** 1 Australian Institute of Marine Science, Townsville, Queensland, Australia; 2 University of Tasmania Institute for Marine and Antarctic Studies, Hobart, Tasmania, Australia; 3 Australian Centre for Ecogenomics, The University of Queensland, Saint Lucia, Queensland, Australia; 4 GEOMAR Helmholtz Centre for Ocean Research Kiel, Kiel, Germany; 5 Appalachian State University, Boone, North Carolina, USA; 6 Tree of Life, Wellcome Sanger Institute, Hinxton, England, UK; 7 University of New South Wales School of Biological Earth and Environmental Sciences, Sydney, New South Wales, Australia

**Keywords:** Cliona cf. orientalis, sponge, genome sequence, chromosomal, Clionaida, microbial metagenome

## Abstract

We present a genome assembly from a specimen of *Cliona cf. orientalis* (Porifera; Demospongiae; Clionaida; Clionaidae). The genome sequence has a total length of 217.17 megabases. Most of the assembly (98.28%) is scaffolded into 19 chromosomal pseudomolecules. The mitochondrial genome has also been assembled and is 19.63 kilobases in length. Gene annotation of this assembly on Ensembl identified 25,502 protein-coding genes. Furthermore, three prokaryotic binned genomes were generated, including a high-quality metagenome-assembled genome (MAG) of the family Parvibaculaceae. Although Symbiodiniaceae sequences were also identified, a complete genome assembly could not be generated due to low coverage.

## Species taxonomy

Eukaryota; Opisthokonta; Metazoa; Porifera; Demospongiae; Heteroscleromorpha; Clionaida; Clionaidae; *Cliona*; *Cliona cf. orientalis* Thiele, 1900 (NCBI:txid281731). 

## Background

The demosponge *Cliona orientalis* (Thiele, 1900) is widely distributed in the Indo-Pacific region, where it accelerates coral reef erosion due to its significant bioeroding capacity ( Schönberg *et al.*, 2017). Like other members of the *Cliona viridis* species complex, *C. orientalis* is associated with intracellular dinoflagellates of the family Symbiodiniaceae ( LaJeunesse *et al.*, 2018; Ramsby *et al.*, 2018a), whose photochemical activity promotes the energetically demanding excavation rates of the host ( Achlatis *et al.*, 2021). In *C. orientalis*, the Symbiodiniaceae community is highly conserved across geographic regions, and consists primarily of *Gerakladium endoclionum*, which belongs to one of the more ancestral Symbiodiniaceae lineages ( Hill *et al.*, 2011; LaJeunesse *et al.*, 2018; Ramsby *et al.*, 2017; Ramsby *et al.*, 2018a; Schönberg & Loh, 2005). The Symbiodiniaceae play a key role in the direct assimilation of inorganic carbon and nitrogen within this phototrophic sponge, as well as in the recycling of host nitrogenous waste ( Achlatis *et al.*, 2018; Achlatis *et al.*, 2019). In addition to algal symbionts, *C. orientalis* harbours a sparse prokaryotic community dominated by Alphaproteobacteria ( Ramsby *et al.*, 2018b). A recent metagenomic study indicates that the dominant prokaryotes associated with *C. orientalis* are affiliated to Parvibaculales ( Robbins *et al.*, 2021); notably, bacteria belonging to the family Parvibaculaceae were found associated with cultured Symbiodiniaceae and possess genes that could be involved in bacterial-algal interactions, such as a metabolic potential for vitamin B _12 _biosynthesis ( Shoguchi *et al.*, 2024). However, the prokaryotic contribution to *Cliona* metabolic processes appears to be limited, at least in the outer sponge layer ( Achlatis *et al.*, 2018; Achlatis *et al.*, 2019).

Similar to cnidarian-Symbiodiniaceae associations, the symbiosis is destabilised under severe thermal stress, leading to the onset of bleaching and mortality ( Ramsby *et al.*, 2018a). Remarkably, sponge-Symbiodiniaceae relationships show higher thermotolerance compared to coral symbioses ( Ramsby *et al.*, 2018a; Schönberg *et al.*, 2008), suggesting that distinct molecular mechanisms may govern these mutualistic interactions. Hence, the genome assembly of *C. cf. orientalis* and the sequence data of its dinoflagellate endosymbiont *G. endoclionum* provides an unprecedented opportunity for better understanding the molecular basis underlying the adaptive capacity of this resilient symbiosis, and elucidating the ecology and evolution of host-Symbiodiniaceae relationships.

Furthermore, the genome of *C. cf. orientalis* represents a valuable resource for enhancing our understanding of the molecular mechanisms underpinning sponge bioeroding capacity. Recent studies on *Cliona* species belonging to the *C. viridis* complex suggest that the dissolution of calcium carbonate (CaCO _3_) is promoted by the release of protons from intracellular vesicles at the etching sites, and that carbonic anhydrase enzymes may convert the resulting bicarbonate ions to carbon dioxide, which may be utilized by the sponge-associated Symbiodiniaceae ( Achlatis *et al.*, 2021; Webb *et al.*, 2019). However, the mechanisms governing the active transport of protons, as well as the removal of calcium ions from the dissolution site, have yet to be fully elucidated ( Achlatis *et al.*, 2021; Sullivan & Faulkner, 1990; Webb *et al.*, 2019). Following the chemical dissolution of CaCO _3_, the CaCO _3 _chips are mechanically removed ( Zundelevich *et al.*, 2007), possibly via tissue coordination pathways formed by myocyte-like cells ( Webb *et al.*, 2019). The *Cliona* genome holds the potential to provide key insights into the regulation of both the chemical and mechanical mechanisms involved in the complex process of bioerosion.

## Genome sequence report

### Sequencing data

**Figure 1.**
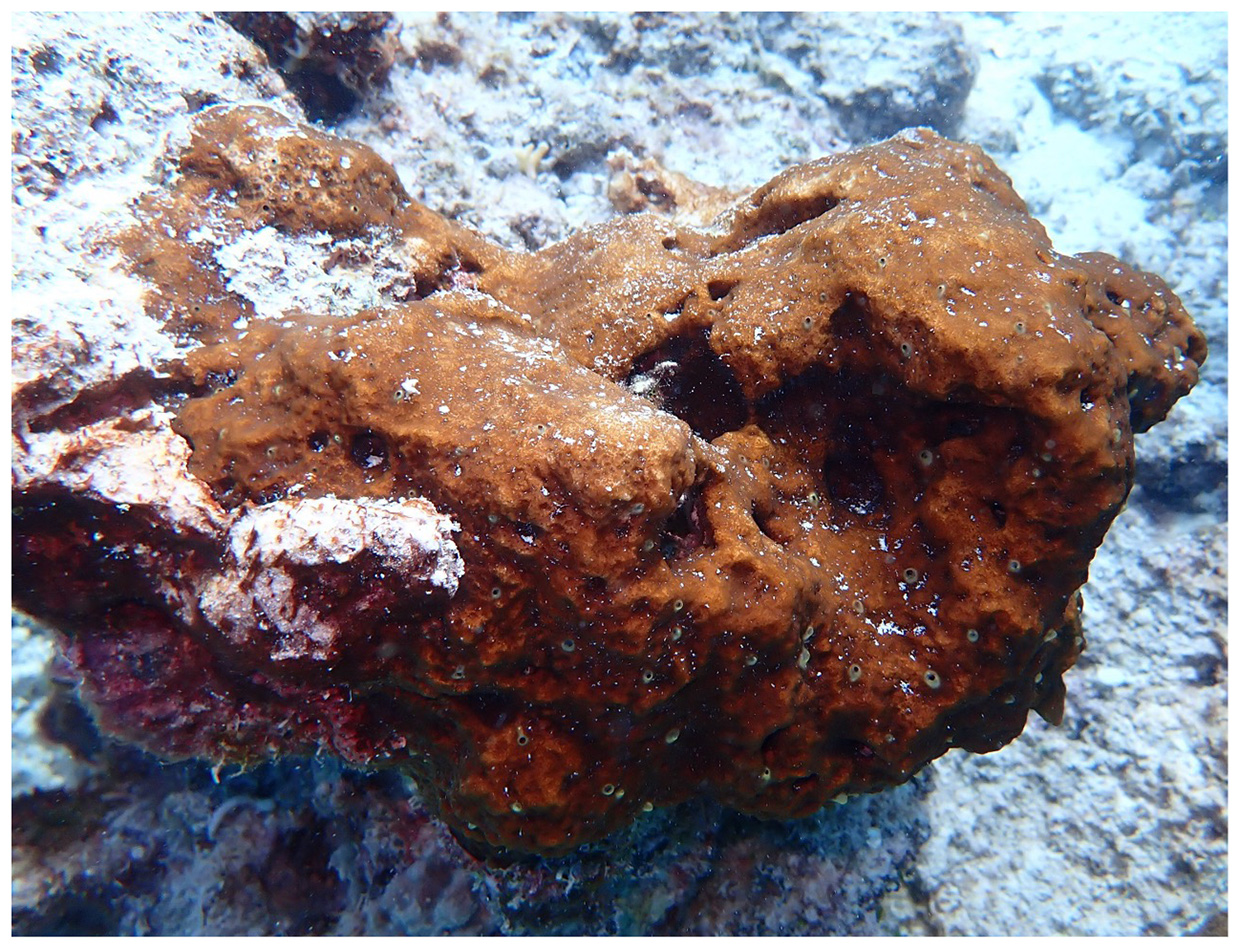
In-situ image of the *Cliona cf. orientalis* individual (odCliOrie1) used for sequencing. The individual was collected from Davies Reef on the Great Barrier Reef.

The genome of a specimen of *Cliona cf. orientalis* ( Figure 1) was sequenced using Pacific Biosciences single-molecule HiFi long reads, generating 38.47 Gb (gigabases) from 4.11 million reads. Based on the estimated genome size, the sequencing data provided approximately 90.0x coverage of the host genome. Chromosome conformation Hi-C data produced 106.35 Gb from 704.32 million reads. Table 1 summarises the specimen and sequencing information.

**Table 1.** Specimen and sequencing data for *Cliona cf. orientalis*.

Project information			
**Study title**	Cliona orientalis		
**Umbrella BioProject**	PRJEB65616		
**Species**	*Cliona orientalis*		
**BioSpecimen**	SAMEA9614640		
**NCBI taxonomy ID**	281731		
Specimen information			
**Technology**	**ToLID**	**BioSample accession**	**Organism part**
**PacBio long read sequencing**	odCliOrie1	SAMEA9614697	Somatic tissue
**Hi-C sequencing**	odCliOrie1	SAMEA9614702	Somatic tissue
**RNA sequencing**	odCliOrie1	SAMEA9614702	Somatic tissue
Sequencing information			
**Platform**	**Run accession**	**Read count**	**Base count (Gb)**
**Hi-C Illumina NovaSeq 6000**	ERR12512720	7.04e+08	106.35
**PacBio Revio**	ERR12015694	2.40e+06	22.11
**PacBio Sequel IIe**	ERR12408767	1.72e+06	16.36
**RNA Illumina NovaSeq X**	ERR13669944	1.16e+08	17.47

### Assembly statistics

The primary haplotype was assembled, and contigs corresponding to an alternate haplotype were also deposited in INSDC databases. The assembly was improved by manual curation, which corrected 201 misjoins or missing joins and removed 81 haplotypic duplications. These interventions reduced the total assembly length by 32.33%, decreased the scaffold count by 77.49%, and also decreased the scaffold N50 by 1.89%. The final assembly has a total length of 217.17 Mb in 307 scaffolds, with 216 gaps, and a scaffold N50 of 9.97 Mb ( Table 2).

**Table 2.** Genome assembly data for *Cliona cf. orientalis*.

Genome assembly		
Assembly name	odCliOrie1.1	
Assembly accession	GCA_963930775.1	
*Alternate haplotype accession*	*GCA_963930685.1*	
Assembly level for primary assembly	chromosome	
Span (Mb)	217.17	
Number of contigs	523	
Number of scaffolds	307	
Longest scaffold (Mb)	37.5	
**Assembly metric**	**Measure**	*Benchmark*
Contig N50 length	1.98 Mb	*≥ 1 Mb*
Scaffold N50 length	9.97 Mb	*= chromosome N50*
Consensus quality (QV)	Primary: 58.0; alternate: 47.9; combined 48.5	*≥ 40*
*k*-mer completeness	Primary: 60.50%; alternate: 91.43%; combined: 99.62%	*≥ 95%*
BUSCO *	C:80.8%[S:79.4%,D:1.4%], F:7.2%,M:11.9%,n:954	*S > 90%, D < 5%*
Percentage of assembly mapped to chromosomes	98.3%	*≥ 90%*
Organelles	Mitochondrial genome: 19.63 kb	*complete single alleles*
Genome annotation of assembly GCA_963930775.1 at Ensembl		
Number of protein-coding genes	25,502	
Number of non-coding genes	5,705	
Number of gene transcripts	41,693	

* BUSCO scores based on the metazoa_odb10 BUSCO set using version 5.5.0. C = complete [S = single copy, D = duplicated], F = fragmented, M = missing, n = number of orthologues in comparison.

**Figure 2.**
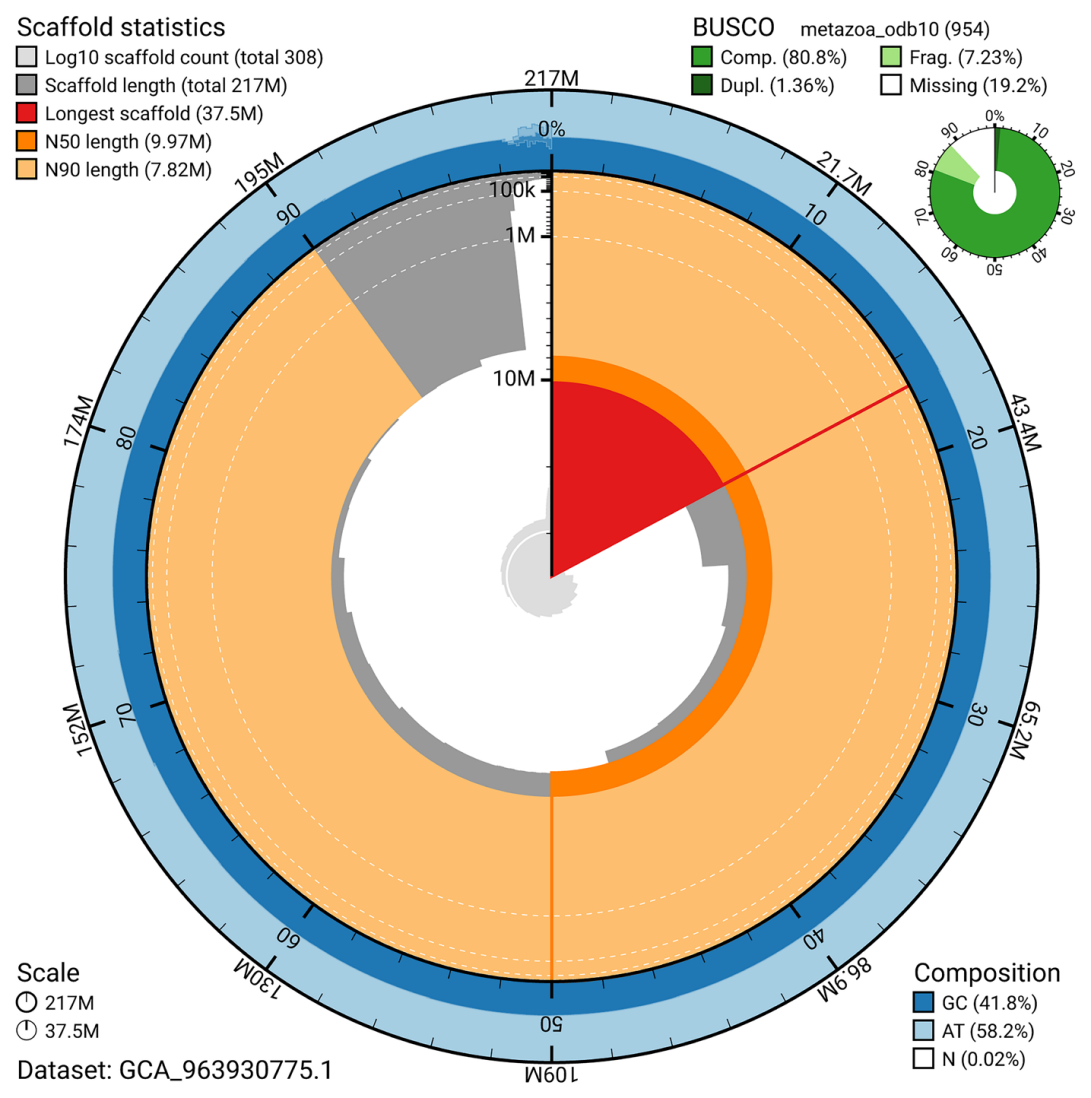
Genome assembly of *Cliona cf. orientalis*, odCliOrie1.1: metrics. The BlobToolKit snail plot provides an overview of assembly metrics and BUSCO gene completeness. The circumference represents the length of the whole genome sequence, and the main plot is divided into 1,000 bins around the circumference. The outermost blue tracks display the distribution of GC, AT, and N percentages across the bins. Scaffolds are arranged clockwise from longest to shortest and are depicted in dark grey. The longest scaffold is indicated by the red arc, and the deeper orange and pale orange arcs represent the N50 and N90 lengths. A light grey spiral at the centre shows the cumulative scaffold count on a logarithmic scale. A summary of complete, fragmented, duplicated, and missing BUSCO genes in the set is presented at the top right. An interactive version of this figure is available at https://blobtoolkit.genomehubs.org/view/GCA_963930775.1/dataset/GCA_963930775.1/snail.

**Figure 3.**
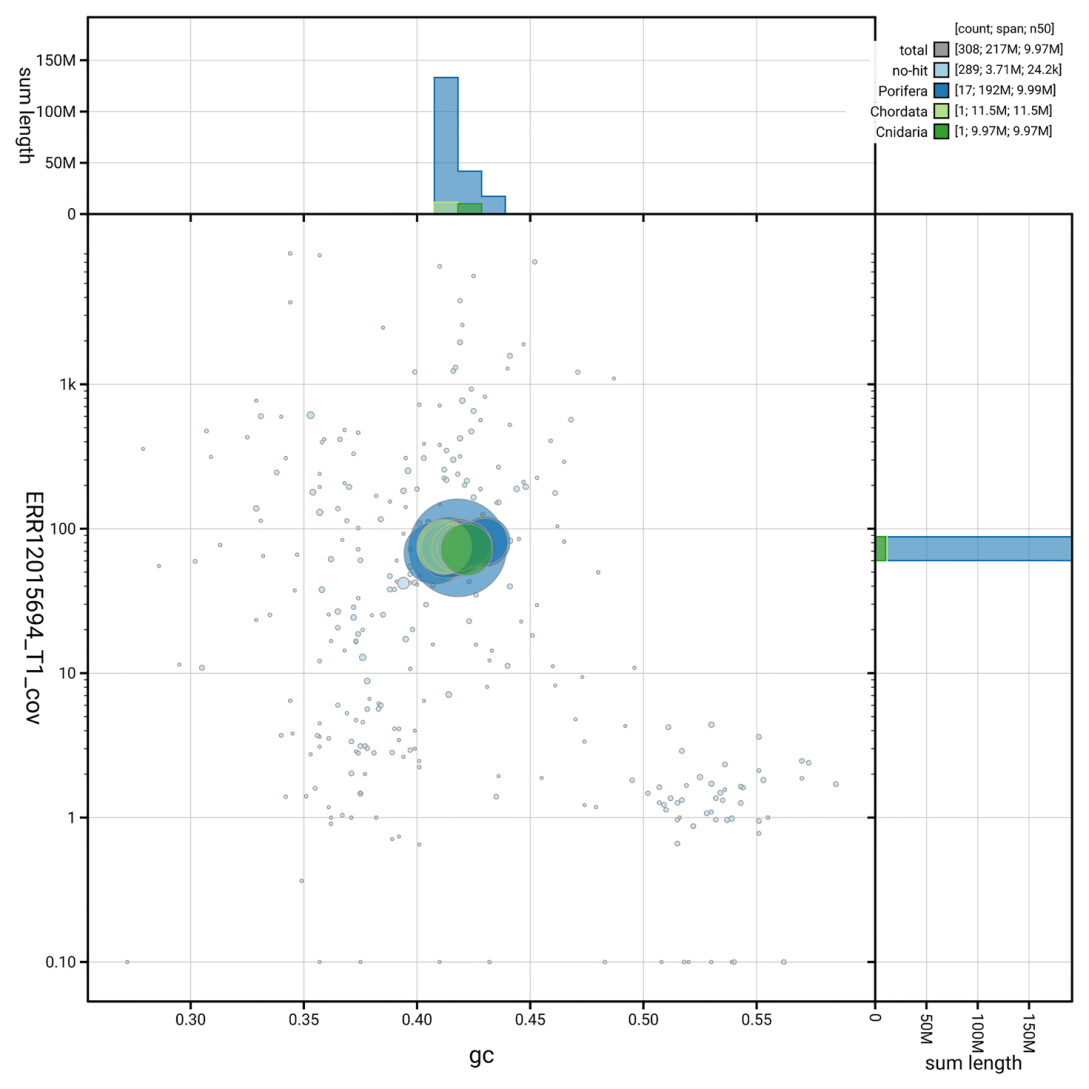
Genome assembly of *Cliona cf. orientalis*, odCliOrie1.1: BlobToolKit GC-coverage plot. Blob plot showing sequence coverage (vertical axis) and GC content (horizontal axis). The circles represent scaffolds, with the size proportional to scaffold length and the colour representing phylum membership. The histograms along the axes display the total length of sequences distributed across different levels of coverage and GC content. An interactive version of this figure is available at https://blobtoolkit.genomehubs.org/view/GCA_963930775.1/dataset/GCA_963930775.1/blob.

**Figure 4.**
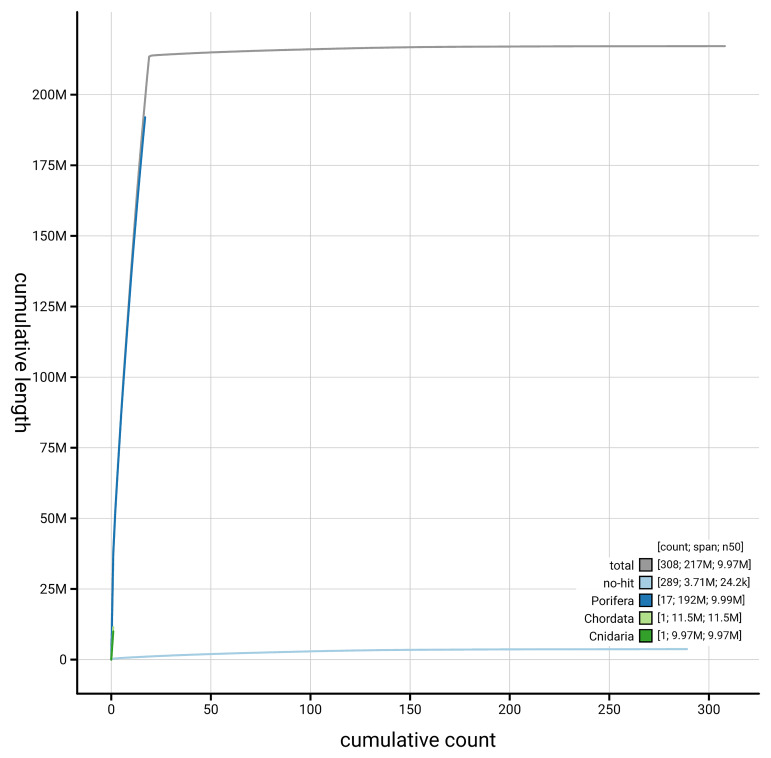
Genome assembly of *Cliona cf. orientalis,* odCliOrie1.1: BlobToolKit cumulative sequence plot. The grey line shows cumulative length for all scaffolds. Coloured lines show cumulative lengths of scaffolds assigned to each phylum using the buscogenes taxrule. An interactive version of this figure is available at https://blobtoolkit.genomehubs.org/view/GCA_963930775.1/dataset/GCA_963930775.1/cumulative.

The snail plot in Figure 2 provides a summary of the assembly statistics, indicating the distribution of scaffold lengths and other assembly metrics. Figure 3 shows the distribution of scaffolds by GC proportion and coverage. Figure 4 presents a cumulative assembly plot, with separate curves representing different scaffold subsets assigned to various phyla, illustrating the completeness of the assembly.

**Figure 5.**
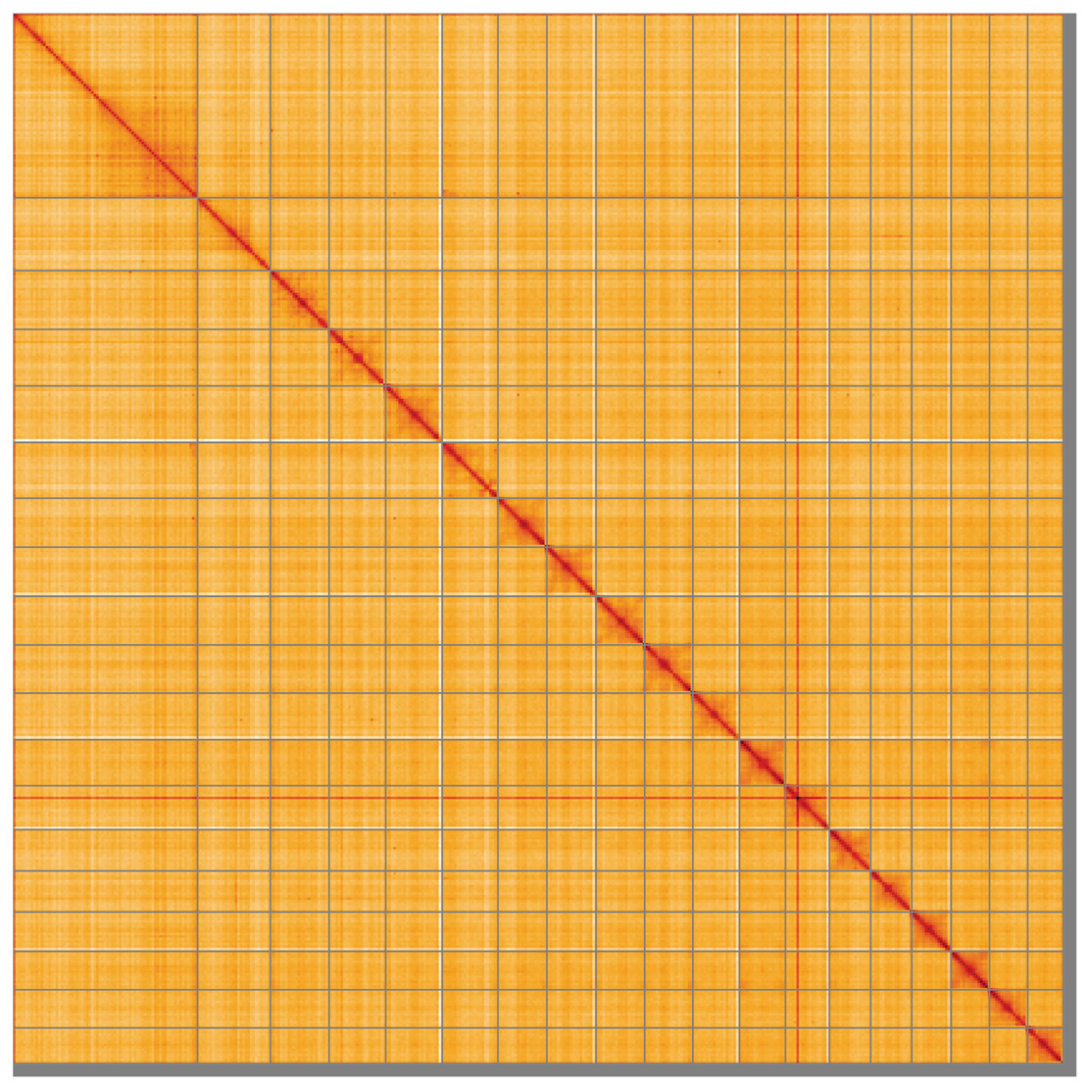
Genome assembly of *Cliona cf. orientalis*: Hi-C contact map of the odCliOrie1.1 assembly, visualised using HiGlass. Chromosomes are shown in order of size from left to right and top to bottom. An interactive version of this figure may be viewed at https://genome-note-higlass.tol.sanger.ac.uk/l/?d=I1ESe8w9TSqDpEz7j8Lrrw.

Most of the assembly sequence (98.3%) was assigned to 19 chromosomal-level scaffolds. These chromosome-level scaffolds, confirmed by Hi-C data, are named according to size ( Figure 5; Table 3).

**Table 3.** Chromosomal pseudomolecules in the genome assembly of *Cliona cf. orientalis*, odCliOrie1.

INSDC accession	Name	Length (Mb)	GC%
OZ005689.1	1	37.5	42
OZ005690.1	2	14.8	41
OZ005691.1	3	11.96	41.5
OZ005692.1	4	11.51	41
OZ005693.1	5	11.46	41.5
OZ005694.1	6	11.33	41.5
OZ005695.1	7	9.99	42
OZ005696.1	8	9.97	42
OZ005697.1	9	9.89	41.5
OZ005698.1	10	9.79	42
OZ005699.1	11	9.46	41.5
OZ005700.1	12	9.36	43
OZ005701.1	13	8.93	42
OZ005702.1	14	8.4	42
OZ005703.1	15	8.35	41.5
OZ005704.1	16	7.98	42.5
OZ005705.1	17	7.82	43
OZ005706.1	18	7.76	42
OZ005707.1	19	7.22	42.5
OZ005708.1	MT	0.02	34

The mitochondrial genome was also assembled. This sequence is included as a contig in the multifasta file of the genome submission and as a standalone record in GenBank.

While sequence data associated with the Symbiodiaceae symbiont were found in the sample, low coverage (~2×) precluded the generation of a complete assembly.

### Assembly quality metrics

The primary haplotype has a QV of 58.0, and the combined primary and alternate assemblies achieve an estimated QV of 48.5 ( Table 2). The *k*-mer completeness for the primary haplotype is 60.50%, and for the alternate haplotype it is 91.43%. The combined primary and alternate assemblies achieve a *k*-mer completeness of 99.62% ( Table 2). BUSCO v.5.5.0 analysis using the metazoa_odb10 reference set ( *n* = 954) indicated a completeness score of 80.8% (single = 79.4%, duplicated = 1.4%; Table 2).

## Metagenome report

**Figure 6.**
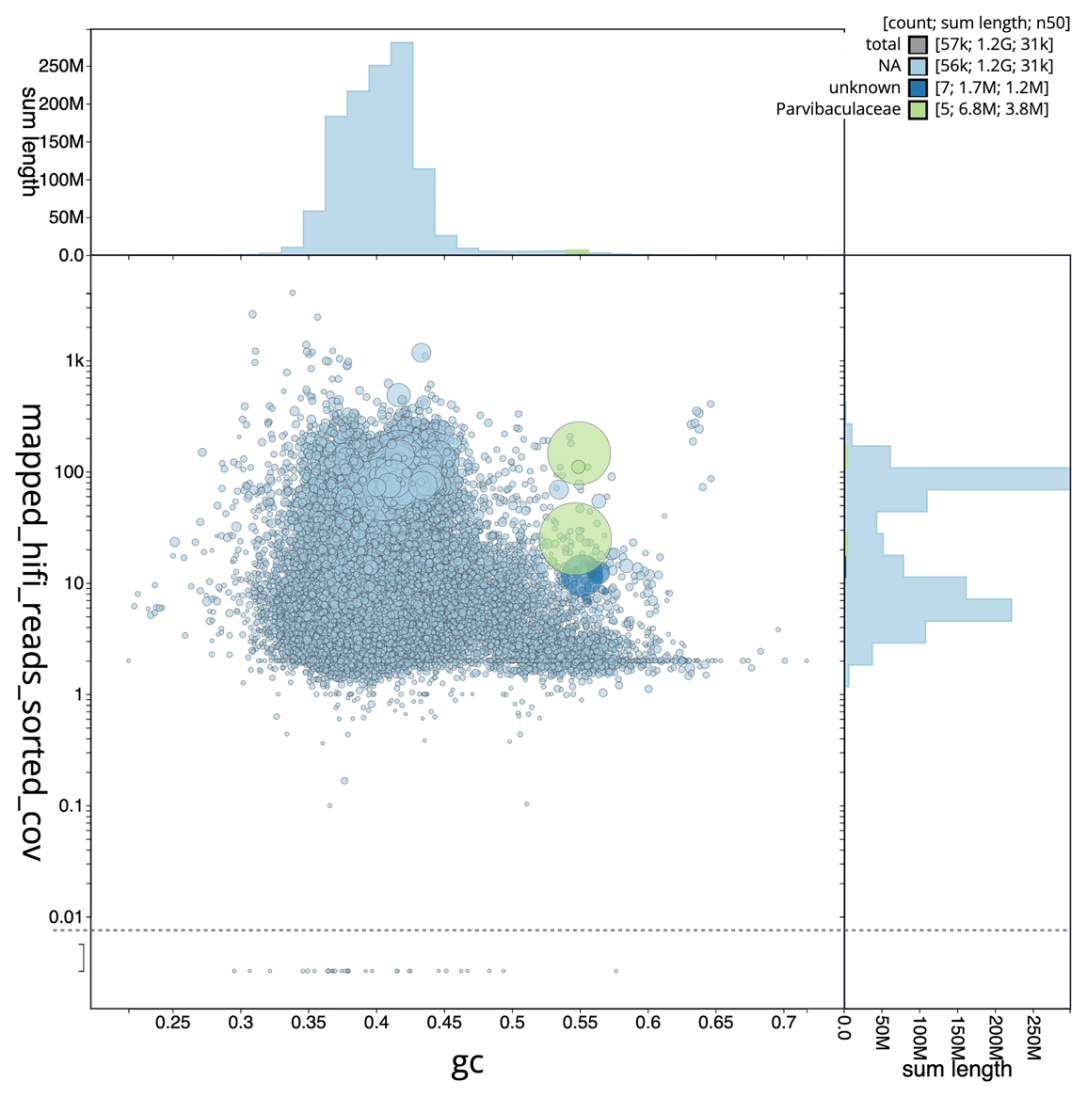
Blob plot of base coverage in mapped against GC proportion for sequences in the *Cliona cf. orientalis* metagenome. Binned contigs are coloured by family. Circles are sized in proportion to sequence length on a square root scale, ranging from 510 to 5,997,640. Histograms show the distribution of sequence length sum along each axis. An interactive version of this figure may be viewed here.

**Figure 7.**
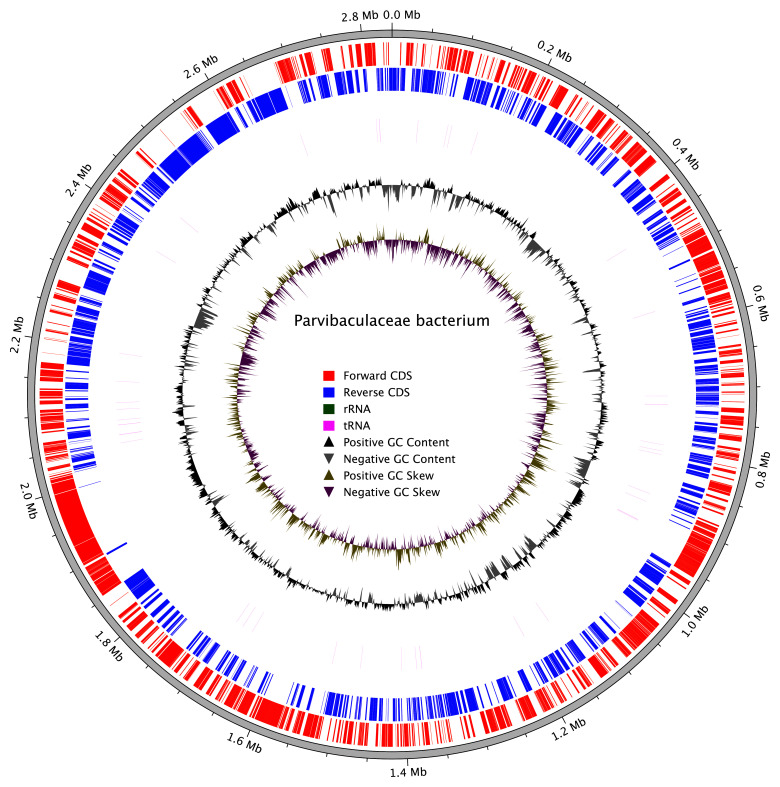
Circular genome map of the Parvibaculaceae bacterium MAG. The outer rings represent coding sequences (CDS) on the forward (red) and reverse (blue) strands, with rRNA (green) and tRNA (purple) annotations. The inner rings depict GC content (black) and GC skew (green and purple), indicating variations in nucleotide composition across the genome.

Three binned genomes were generated from the metagenome assembly ( Figure 6), of which one, a Parvibaculaceae bacterium ( Figure 7), was classified as a high-quality metagenome assembled genome (MAG) (see methods). For details on binned genomes see Table 4.

**Table 4.** Quality metrics and taxonomic assignments of the metagenome sequences.

NCBI taxon	Taxid	GTDB taxonomy	Quality	Size (bp)	Contigs	Circular	Mean coverage	Completeness (%)	Contamination (%)
Thermodesulfobacteriota bacterium	2202153	g__GCA-014075295	Medium	1,686,746	7	No	6.43	87.82	4.62
Parvibaculaceae bacterium	2813037	f__RS24	Medium	4,006,238	4	No	13	90.92	6.82
Parvibaculaceae bacterium	2813037	f__RS24	High	2,895,071	1	Yes	86.76	92.44	0.45

## Genome annotation report

The *Cliona cf. orientalis* genome assembly (GCA_963930775.1) was annotated at the European Bioinformatics Institute (EBI) on Ensembl Rapid Release. The resulting annotation includes 41,693 transcribed mRNAs from 25,502 protein-coding and 5,705 non-coding genes ( Table 2; https://rapid.ensembl.org/Cliona_orientalis_GCA_963930775.1/Info/Index). The average transcript length is 5,724.28. There are 1.34 coding transcripts per gene and 5.78 exons per transcript.

## Methods

### Sample acquisition

A specimen of *Cliona cf. orientalis* specimen ID GHC0000115, ToLID odCliOrie1; Figure 1) was collected from Davies Reef on the Great Barrier Reef (latitude 18.91, longitude 147.7) on 2020-09-23 by SCUBA diving. The specimen was transported in a shaded aquarium to the National Sea Simulator at the Australian Institute of Marine Science (Townsville, Australia) where it was snap frozen in liquid nitrogen and stored at –80°C until further processing. 

### Nucleic acid extraction

The workflow for high molecular weight (HMW) DNA extraction at the Wellcome Sanger Institute (WSI) Tree of Life Core Laboratory includes a sequence of procedures: sample preparation and homogenisation, DNA extraction, fragmentation and purification. Detailed protocols are available on protocols.io ( Denton *et al.*, 2023). Prior to DNA extraction, the sponge sample was bathed in “L buffer” (10 mM Tris, pH 7.6, 100 mM EDTA, 20 mM NaCl), minced into small pieces using a scalpel and the cellular interior separated from the mesohyl using forceps ( Lopez, 2022).

HMW DNA was extracted using the Automated MagAttract v2 protocol ( Oatley *et al.*, 2023a). For ULI PacBio sequencing, DNA was fragmented using the Covaris g-TUBE method ( Oatley *et al.*, 2023c). Sheared DNA was purified by solid-phase reversible immobilisation, using AMPure PB beads to eliminate shorter fragments and concentrate the DNA ( Oatley *et al.*, 2023b). The concentration of the sheared and purified DNA was assessed using a Nanodrop spectrophotometer and Qubit Fluorometer using the Qubit dsDNA High Sensitivity Assay kit. Fragment size distribution was evaluated by running the sample on the FemtoPulse system.

RNA was extracted from tissue of odCliOrie1 in the Tree of Life Laboratory at the WSI using the RNA Extraction: Automated MagMax™ *mir*Vana protocol ( do Amaral *et al.*, 2023). The RNA concentration was assessed using a Nanodrop spectrophotometer and a Qubit Fluorometer using the Qubit RNA Broad-Range Assay kit. Analysis of the integrity of the RNA was done using the Agilent RNA 6000 Pico Kit and Eukaryotic Total RNA assay.

### Sequencing

Pacific Biosciences HiFi circular consensus DNA sequencing libraries were prepared according to the manufacturer’s instructions. Poly(A) RNA-Seq libraries were prepared using the NEB Ultra II RNA Library Prep Kit. DNA sequencing was carried out by the WSI Scientific Operations core using Pacific Biosciences Sequel IIe and Revio instruments (HiFi), while RNA sequencing was performed on anIllumina NovaSeq X instrument. Tissue from odCliOrie1 was processed using the Arima2 kit, and Hi-C data were generated by sequencing on the Illumina NovaSeq 6000.

### Genome assembly, curation and evaluation

***Host assembly and curation***

The HiFi reads were assembled using Hifiasm ( Cheng *et al.*, 2021) with the --primary option. Haplotypic duplications were identified and removed using purge_dups ( Guan *et al.*, 2020). The Hi-C reads were mapped to the primary contigs using bwa-mem2 ( Vasimuddin *et al.*, 2019). The contigs were further scaffolded using the provided Hi-C data ( Rao *et al.*, 2014) in YaHS ( Zhou *et al.*, 2023) using the --break option for handling potential misassemblies. The scaffolded assemblies were evaluated using Gfastats ( Formenti *et al.*, 2022), BUSCO ( Manni *et al.*, 2021) and MERQURY.FK ( Rhie *et al.*, 2020).

The mitochondrial genome was assembled using MitoHiFi ( Uliano-Silva *et al.*, 2023), which runs MitoFinder ( Allio *et al.*, 2020) and uses these annotations to select the final mitochondrial contig and to ensure the general quality of the sequence.

The assembly was decontaminated using the Assembly Screen for Cobionts and Contaminants (ASCC) pipeline. Flat files and maps used in curation were generated via the TreeVal pipeline ( Pointon *et al.*, 2023). Manual curation was conducted primarily in PretextView ( Harry, 2022) and HiGlass ( Kerpedjiev *et al.*, 2018), with additional insights provided by JBrowse2 ( Diesh *et al.*, 2023). Scaffolds were visually inspected and corrected as described by Howe *et al.* (2021). Any identified contamination, missed joins, and mis-joins were amended, and duplicate sequences were tagged and removed. The curation process is documented at https://gitlab.com/wtsi-grit/rapid-curation.

***Host assembly quality assessment***

The Merqury.FK tool ( Rhie *et al.*, 2020), run in a Singularity container ( Kurtzer *et al.*, 2017), was used to evaluate *k*-mer completeness and assembly quality for the primary and alternate haplotypes using the *k*-mer databases ( *k* = 31) that were computed prior to genome assembly.

A Hi-C contact map was produced for the final version of the assembly. The Hi-C reads were aligned using bwa-mem2 ( Vasimuddin *et al.*, 2019) and the alignment files were combined using SAMtools ( Danecek *et al.*, 2021). The Hi-C alignments were converted into a contact map using BEDTools ( Quinlan & Hall, 2010) and the Cooler tool suite ( Abdennur & Mirny, 2020). The contact map is visualised in HiGlass ( Kerpedjiev *et al.*, 2018).

The blobtoolkit pipeline is a Nextflow port of the previous Snakemake Blobtoolkit pipeline ( Challis *et al.*, 2020). It aligns the PacBio reads in SAMtools and minimap2 ( Li, 2018) and generates coverage tracks for regions of fixed size. In parallel, it queries the GoaT database ( Challis *et al.*, 2023) to identify all matching BUSCO lineages to run BUSCO ( Manni *et al.*, 2021). For the three domain-level BUSCO lineages, the pipeline aligns the BUSCO genes to the UniProt Reference Proteomes database ( Bateman *et al.*, 2023) with DIAMOND blastp ( Buchfink *et al.*, 2021). The genome is also divided into chunks according to the density of the BUSCO genes from the closest taxonomic lineage, and each chunk is aligned to the UniProt Reference Proteomes database using DIAMOND blastx. Genome sequences without a hit are chunked using seqtk and aligned to the NT database with blastn ( Altschul *et al.*, 1990). The blobtools suite combines all these outputs into a blobdir for visualisation.

The blobtoolkit pipeline was developed using nf-core tooling ( Ewels *et al.*, 2020) and MultiQC ( Ewels *et al.*, 2016), relying on the Conda package manager, the Bioconda initiative ( Grüning *et al.*, 2018), the Biocontainers infrastructure ( da Veiga Leprevost *et al.*, 2017), as well as the Docker ( Merkel, 2014) and Singularity ( Kurtzer *et al.*, 2017) containerisation solutions.

Table 5 contains a list of relevant software tool versions and sources.

**Table 5.** Software tools: versions and sources.

Software tool	Version	Source
BEDTools	2.30.0	https://github.com/arq5x/bedtools2
BLAST	2.14.0	ftp://ftp.ncbi.nlm.nih.gov/blast/executables/blast+/
BlobToolKit	4.3.3	https://github.com/blobtoolkit/blobtoolkit
BUSCO	5.5.0	https://gitlab.com/ezlab/busco
bwa-mem2	2.2.1	https://github.com/bwa-mem2/bwa-mem2
CheckM	1.2.1	https://github.com/Ecogenomics/CheckM
Cooler	0.8.11	https://github.com/open2c/cooler
DIAMOND	2.1.8	https://github.com/bbuchfink/diamond
dRep	3.4.0	https://github.com/MrOlm/drep
fasta_windows	0.2.4	https://github.com/tolkit/fasta_windows
FastK	427104ea91c78c3b8b8b49f1a7d6bbeaa869ba1c	https://github.com/thegenemyers/FASTK
Gfastats	1.3.6	https://github.com/vgl-hub/gfastats
GoaT CLI	0.2.5	https://github.com/genomehubs/goat-cli
GTDB-TK	2.3.2	https://github.com/Ecogenomics/GTDBTk
Hifiasm	0.19.5-r587	https://github.com/chhylp123/hifiasm
HiGlass	44086069ee7d4d3f6f3f0012569789ec138f42b84aa44357826c0b6753eb28de	https://github.com/higlass/higlass
MAGScoT	1.0.0	https://github.com/ikmb/MAGScoT
MerquryFK	d00d98157618f4e8d1a9190026b19b471055b22e	https://github.com/thegenemyers/MERQURY.FK
MetaBat2	2.15-15-gd6ea400	https://bitbucket.org/berkeleylab/metabat/src/master/
metaMDBG	-	https://github.com/GaetanBenoitDev/metaMDBG
Minimap2	2.24-r1122	https://github.com/lh3/minimap2
MitoHiFi	2	https://github.com/marcelauliano/MitoHiFi
MultiQC	1.14, 1.17, and 1.18	https://github.com/MultiQC/MultiQC
Nextflow	23.04.1	https://github.com/nextflow-io/nextflow
PretextView	0.2	https://github.com/sanger-tol/PretextView
PROKKA	1.14.5	https://github.com/vdejager/prokka
purge_dups	1.2.3	https://github.com/dfguan/purge_dups
samtools	1.18	https://github.com/samtools/samtools
sanger-tol/ascc	-	https://github.com/sanger-tol/ascc
sanger-tol/ blobtoolkit	0.3.0	https://github.com/sanger-tol/blobtoolkit
Seqtk	1.3	https://github.com/lh3/seqtk
Singularity	3.9.0	https://github.com/sylabs/singularity
TreeVal	1.2.0	https://github.com/sanger-tol/treeval
YaHS	1.1a.2	https://github.com/c-zhou/yahs

***Metagenome assembly***

The metagenome assembly was generated using metaMDBG ( Benoit *et al.*, 2024) and binned using MetaBAT2 ( Kang *et al.*, 2019). The resulting bin sets were optimised and collectively refined using DAS Tools. PROKKA ( Seemann, 2014) was used to identify tRNAs and rRNAs in each bin, CheckM was used to assess bin completeness/contamination, and GTDB-TK ( Chaumeil *et al.*, 2022) was used to taxonomically classify bins. Taxonomic replicate bins were identified using dRep ( Olm *et al.*, 2017). All bins were assessed for quality and categorised as metagenome-assembled genomes (MAGs) if they met the following criteria: contamination ≤ 5%, presence of 5S, 16S, and 23S rRNA genes, at least 18 unique tRNAs, and either ≥ 90% completeness or ≥ 50% completeness with fully circularised chromosomes. Bins that did not meet these thresholds, or were identified as taxonomic replicates of MAGs, were retained as ‘binned metagenomes’ provided they had ≥ 50% completeness and ≤ 10% contamination. Software tool versions and sources are given in Table 5.

The closed genome of the Parvibaculaceae MAG was annotated using Prokka ( Seemann, 2014) with default parameters against the UniRef database.

### Genome annotation

The Ensembl Genebuild annotation system ( Aken *et al.*, 2016) was used to generate annotation for the *Cliona cf. orientalis* assembly (GCA_963930775.1) in Ensembl Rapid Release at the EBI. Annotation was created primarily through alignment of transcriptomic data to the genome, with gap filling via protein-to-genome alignments of a select set of proteins from UniProt ( UniProt Consortium, 2019).

### Wellcome Sanger Institute – Legal and Governance

The materials that have contributed to this genome note have been supplied by a Tree of Life collaborator. The Wellcome Sanger Institute employs a process whereby due diligence is carried out proportionate to the nature of the materials themselves, and the circumstances under which they have been/are to be collected and provided for use. The purpose of this is to address and mitigate any potential legal and/or ethical implications of receipt and use of the materials as part of the research project, and to ensure that in doing so we align with best practice wherever possible. The overarching areas of consideration are:

•    Ethical review of provenance and sourcing of the material

•    Legality of collection, transfer and use (national and international)

Each transfer of samples is undertaken according to a Research Collaboration Agreement or Material Transfer Agreement entered into by the Tree of Life collaborator, Genome Research Limited (operating as the Wellcome Sanger Institute) and in some circumstances other Tree of Life collaborators.

## Data Availability

European Nucleotide Archive: Cliona orientalis. Accession number PRJEB65616; https://identifiers.org/ena.embl/PRJEB65616. The genome sequence is released openly for reuse. The *Cliona cf. orientalis* genome sequencing initiative is part of the Aquatic Symbiosis Genomics (ASG) project ( https://www.ebi.ac.uk/ena/browser/view/PRJEB43743). All raw sequence data and the assembly have been deposited in INSDC databases. Raw data and assembly accession identifiers are reported in Table 1 and Table 2.

## Author information

Members of the Wellcome Sanger Institute Tree of Life Management, Samples and Laboratory Team are listed here: https://doi.org/10.5281/zenodo.10066175.

Members of the Wellcome Sanger Institute Scientific Operations: Sequencing Operations are listed here: https://doi.org/10.5281/zenodo.10043364.

Members of the Wellcome Sanger Institute Tree of Life Core Informatics team are listed here: https://doi.org/10.5281/zenodo.10066637.

Members of the European Bioinformatics Institute ASG Data Portal team are listed here: https://doi.org//10.5281/zenodo.10076466.

Members of the Wellcome Sanger Institute/Aquatic Symbiosis Genomics Project Leadership are listed here: https://doi.org/10.5281/zenodo.10184833.
