# Supplementary material for: The chromosomal genome sequence of the sponge Cliona cf. orientalis Thiele (1900) and its associated microbial metagenome sequences
Source: Wellcome Open Res. 2025 Jul 9;10:342. [Version 1] doi: 10.12688/wellcomeopenres.24304.1 (PMC12639293; doi:10.12688/wellcomeopenres.24304.1)
Supplement: Peer review history

# Reviewer report: Reviewer response for version 1

**DOI:** 10.21956/wellcomeopenres.26804.r137655

**Published:** 2025-11-22

**Authors:** Richard R Copley

**Affiliations:** 1 Sorbonne Universite, Paris, Île-de-France, France

This paper describes the Tree of Life project genome sequence for the sponge *Cliona cf. orientalis*. It is a fine report and reference for the availability of these data. The usual excellent modern quality is reported, with the genome assembled into 19 pseudo-chromosomes. In addition, data are available for three prokaryotes. A protein dataset is available from Ensembl. The BUSCO metric of gene completeness is ~80% for metazoa_odb10, which seems in line with other recently produced sponge genomes. As such, the data should provide a valuable resource to researchers involved in the field. The authors present some useful background to areas of specific interest regarding C *liona orientalis*, in particular its reef eroding capabilities. The references provided should be a useful inroad for researchers unfamiliar with this area.

Are sufficient details of methods and materials provided to allow replication by others?

Yes

Is the rationale for creating the dataset(s) clearly described?

Yes

Are the datasets clearly presented in a useable and accessible format?

Yes

Are the protocols appropriate and is the work technically sound?

Yes

Reviewer Expertise:

protein and genome research

I confirm that I have read this submission and believe that I have an appropriate level of expertise to confirm that it is of an acceptable scientific standard.

# Reviewer report: Reviewer response for version 1

**DOI:** 10.21956/wellcomeopenres.26804.r137657

**Published:** 2025-11-21

**Authors:** Sulav Indra Paul

**Affiliations:** 1 Institute for Biosecurity and Microbial Forensics, Oklahoma State University Ferguson College of Agriculture, Stillwater, Oklahoma, USA

My comments on this data note are as follows:

1. The background is comprehensive and technically aligned with the manuscript's genomic focus. However, the current knowledge gap and study objective are only implied rather than stated explicitly. Adding clear statement at the end of the introduction that states the purpose would improve scientific precision.
2. While this article is presented as a data note and effectively reports a high-quality chromosomal assembly and associated metagenomes, the biological interpretation is minimal. Expanding on what these genomic findings reveal about *Cliona cf. orientalis*’s bioerosion mechanisms or symbiotic interactions would help readers appreciate the broader significance of the data.
3. To ensure the reproducibility of this study, I suggest briefly mentioning the library preparation protocol used for each sequencing approach.

Are sufficient details of methods and materials provided to allow replication by others?

Partly

Is the rationale for creating the dataset(s) clearly described?

Partly

Are the datasets clearly presented in a useable and accessible format?

Yes

Are the protocols appropriate and is the work technically sound?

Yes

Reviewer Expertise:

Microbial genomics

I confirm that I have read this submission and believe that I have an appropriate level of expertise to confirm that it is of an acceptable scientific standard, however I have significant reservations, as outlined above.

# Reviewer report: Reviewer response for version 1

**DOI:** 10.21956/wellcomeopenres.26804.r137654

**Published:** 2025-11-20

**Authors:** Poppy Hesketh-Best

**Affiliations:** 1 IGTP Institut Germans Trias i Pujol, Barcelona, Spain

In this data note, Marangon *et al.* describe the sequencing and assembly of the whole genome of *Cliona cf. orientalis*, along with the assembly of three bacterial metagenome-assembled genomes (MAGs). The authors clearly articulate the necessity and significance of this genomic resource, which provides valuable insights for researchers studying sponge genomics, host–microbe interactions, and symbiosis.

 While I cannot comment on the technical soundness of the sponge genome assembly, as I lack direct experience with these specific techniques, the methods used for the bacterial metagenome assemblies appear sound and consistent with current, well-established practices. Overall, this dataset represents a useful and important contribution to the field of sponge symbiosis research. My only concern is regarding the availability of the bacterial MAGs, which requires clarification.

**Are sufficient details of methods and materials provided to allow replication by others?**

 The authors have provided detailed information on the software versions and sources used in their analyses (Table 5). For the host assembly, the workflow is clearly described and is publicly available through the BlobToolkit Nextflow pipeline, which supports reproducibility.

 However, the bacterial metagenomic assembly section lacks the same level of methodological detail. To strengthen reproducibility, it would be valuable for the authors to provide the specific working scripts (e.g., R, shell, or Python) used during the analysis to indicate parameter settings. If default parameters were used throughout, this should be explicitly stated.

**Are the datasets clearly presented in a usable and accessible format?**

 The data are partly accessible. I was able to access the ENA accession PRJEB65616 (as of 10 November 2025).

 As mentioned above, my main concern is that the bacterial MAGs do not appear to be publicly accessible. If this is intentional, it raises the question of whether including descriptions of these MAGs in the data note is justified. However, given their clear relevance and potential value to the sponge biology community, I strongly recommend making these MAGs individually available to ensure the dataset’s full utility.

Are sufficient details of methods and materials provided to allow replication by others?

Partly

Is the rationale for creating the dataset(s) clearly described?

Yes

Are the datasets clearly presented in a useable and accessible format?

Partly

Are the protocols appropriate and is the work technically sound?

Yes

Reviewer Expertise:

Microbiology (bacterial and viral), microbial (meta-)genomics, host-pathogen infection-networks, host-microbe symbiosis.

I confirm that I have read this submission and believe that I have an appropriate level of expertise to confirm that it is of an acceptable scientific standard, however I have significant reservations, as outlined above.

# Reviewer report: Reviewer response for version 1

**DOI:** 10.21956/wellcomeopenres.26804.r127957

**Published:** 2025-09-01

**Authors:** Amro Hanora, Samar Abdelrahman

**Affiliations:** 1 Microbiology and Immunology, King Salman international University, Ras Suder City, Egypt; 2 Microbiology, Suez University Faculty of Science, Suez, Suez Governorate, Egypt

The present study investigates the chromosomal genome sequence of the sponge Cliona cf. orientalis Thiele (1900) along with its associated microbial metagenome sequences. While the topic is inherently interesting and relevant to marine biology and microbial ecology, the manuscript, in its current form, lacks clarity, depth, and scientific rigor. Based on my evaluation, I believe the paper may not be suitable for publication.

**My main comments are as follows:**

**Comment** 1: **Lack of Clear Research Aim and Contextual Importance.**

 The authors fail to clearly articulate the aim of their study. There is no emphasis on the significance of understanding the factors influencing freshwater sponges, nor is there a discussion of the broader ecological or evolutionary implications. Additionally, the manuscript does not convey what new knowledge has been gained from this research.

**Comment** 2: **Insufficient Novelty**

 The study does not present a clear novel contribution to the field. The findings appear to reiterate existing knowledge without offering new insights or innovative approaches.

**Comment** 3: **Poor Interpretation of Results**

 The results are presented in a raw and descriptive manner, with minimal effort to interpret or contextualize the findings. There is a lack of critical analysis or discussion that would help readers understand the implications of the data.

Are sufficient details of methods and materials provided to allow replication by others?

Partly

Is the rationale for creating the dataset(s) clearly described?

Partly

Are the datasets clearly presented in a useable and accessible format?

Partly

Are the protocols appropriate and is the work technically sound?

Partly

Reviewer Expertise:

Marine microbiology

We confirm that we have read this submission and believe that we have an appropriate level of expertise to state that we do not consider it to be of an acceptable scientific standard, for reasons outlined above.
